# Exploring the Biosafety Potential of *Haberlea rhodopensis* Friv. *In Vitro* Culture Total Ethanol Extract: A Comprehensive Assessment of Genotoxicity, Mitotoxicity, and Cytotoxicity for Therapeutic Applications

**DOI:** 10.3390/cells13131118

**Published:** 2024-06-28

**Authors:** Bela Vasileva, Natalia Krasteva, Kamelia Hristova-Panusheva, Penyo Ivanov, George Miloshev, Atanas Pavlov, Vasil Georgiev, Milena Georgieva

**Affiliations:** 1 Laboratory of Molecular Genetics, Epigenetics and Longevity, Institute of Molecular Biology “Roumen Tsanev”, Bulgarian Academy of Sciences, 1113 Sofia, Bulgaria; byvasileva@gmail.com (B.V.); p.ves.iv@gmail.com (P.I.); karamolbiol@gmail.com (G.M.); 2 Institute of Biophysics and Biomedical Engineering, Bulgarian Academy of Sciences, 1113 Sofia, Bulgaria; nataly@bio21.bas.bg (N.K.); kpanusheva@biomed.bas.bg (K.H.-P.); 3 Laboratory of Cell Biosystems, Institute of Microbiology, Bulgarian Academy of Sciences, 4000 Plovdiv, Bulgaria; a_pavlov@uft-plovidv.bg; 4 Department of Analytical Chemistry and Physical Chemistry, Technological Faculty, University of Food Technologies, 4002 Plovdiv, Bulgaria

**Keywords:** Lep3 human embryonic fibroblast cells, ROS levels, cell viability, neutral comet assay, mitochondrial structure, cell cycle analysis, longevity medicine, nutraceuticals, *Haberlea rhodopensis*, biosafety

## Abstract

The escalating elderly population worldwide has prompted a surge of interest in longevity medicine. Its goal is to interfere with the speed of ageing by slowing it down or even reversing its accompanying effects. As a field, it is rapidly growing and spreading into different branches. One of these is the use of nutraceuticals as anti-ageing drugs. This field is gaining massive popularity nowadays, as people are shifting towards a more natural approach to life and seeking to use natural products as a source of medicine. The present article focuses on the cellular effect of *Haberlea rhodopensis* Friv. in vitro culture total ethanol extract (HRT), produced by a sustainable biotechnological approach. The extract showed a similar phytochemical profile to plant leaf extract and was rich in primary bioactive ingredients—caffeoyl phenylethanoid glycosides, myconoside, and paucifloside. This study examined the biosafety potential, cytotoxicity, genotoxicity, and mitochondrial activity of the extract using in vitro cultures. The results showed high cell survival rates and minimal cytotoxic effects on Lep3 cells, with no induction of reactive oxygen species nor genotoxicity. Additionally, the extract positively influenced mitochondrial activity, indicating potential benefits for cellular health. The results are promising and show the beneficial effect of HRT without the observation of any adverse effects, which sets the foundation for its further testing and potential therapeutic applications.

## 1. Introduction

The gradual decline of biological and physiological functions a person experiences as he grows older defines the term ageing [1]. From a biological perspective, ageing is characterized by a gradual accumulation of cellular and molecular damage, resulting in diminished mental and physical capabilities, a weaker immune system, and a heightened risk of developing diseases [2]. Many factors contribute to this process and are typically divided into two categories—internal, which encompasses hormonal changes, inflammation, and genetic factors, and external, which includes inappropriate nutrient intake, excess alcohol consumption, smoking, and others [3,4].

A main focus in medicine, particularly anti-ageing diagnostics, is to better understand and, if possible, delay the biological ageing process. So far, 14 hallmarks of ageing have been discovered, 5 of which have recently been added [5] to the original 9, which were established by López-Otin in 2013 [6]. These are genomic instability, stem cell exhaustion, cellular senescence, telomere attrition, loss of proteostasis, mitochondrial dysfunction, altered intercellular communication, deregulated nutrient sensing, and epigenetic alterations; the five new additions are compromised autophagy, altered mechanical properties, inflammation, microbiome disturbance, and splicing dysregulation. Yet, despite the vast research in this field, numerous aspects of ageing remain unresolved.

One way the anti-ageing industry is trying to intervene with ageing is through the use of nutraceuticals. In recent years, there has been a noticeable shift in how people approach their health, focusing on the importance of nutrition and generally living a healthier, eco-friendlier, and plant-based life. As an outcome, interest has been sparked in nutraceuticals, functional foods, and dietary supplements [7,8]. This has led to a growing market of phytochemical-enriched extracts sold in different forms of capsules, powders, gels, and other formulations [9]. Plant-derived molecules like polyphenols, flavonoids, and phenolic acids have been linked to different beneficial health changes, exerting potential therapeutic properties and positively affecting ageing processes [9,10,11,12].

*Haberlea rhodopensis* Friv., a plant belonging to the *Gesneriaceae* family, is native to the Balkan region and is considered a glacial relic plant. It is primarily found in Bulgaria, specifically in the Rhodope Mountains, on Sredna Gora mountain, and in the Central Balkan [13]. *H. rhodopensis* is categorized as a resurrection plant, which is a select group of plants known for their remarkable ability to withstand and tolerate extreme dehydration or desiccation [14]. This plant possesses unique characteristics that make it valuable as a model for studying revitalization mechanisms following severe water loss and as a source of genes associated with regeneration. It is also a medicinal plant. The extracts derived from *H. rhodopensis* contain beneficial ingredients like caffeoyl phenylethanoid glycosides, flavone 8-C-glycosides, flavonoid aglycones, and glycosides, along with phenolic acids, demonstrating promising potential in capturing free radicals and reducing oxidative stress [15]. There is significant interest in *H. rhodopensis*, leading to several scientific studies focusing on isolating and identifying its active components [13]. Additionally, these studies explore the pharmacological effects and potential uses of *H. rhodopensis* as a medicinal plant. The primary effects of *H. rhodopensis* include radioprotective, anti-mutagenic, antioxidant, and anti-ageing properties [16]. Very recently, the whole genome of *H. rhodopensis* has been sequenced, and this will unambiguously lead to new and exciting studies of its physiology and beneficial constituents [17].

As a rare species, presented by small populations at few geographical points, it is protected, and collecting individual plants or parts of *H. rhodopensis* from its natural habitats is strongly forbidden. The micropropagation of in vitro plants [18] and alternative propagation in hydroponic systems and greenhouses [19] were slow and economically ineffective methods with low potential for commercialization. Recently, a highly effective biotechnology technique for the renewable production of *H. rhodopensis* biomass using the large-scale cultivation of differentiated and undifferentiated in vitro cultures has been developed and commercialized [20]. *H. rhodopensis* in vitro cultures showed fast growth and accumulated secondary metabolites in higher concentrations when compared to mother plants. Growing *H. rhodopensis* in vitro cultures under controlled conditions is currently the most viable way to sustain the large-scale production of rare metabolites such as myconoside. However, only rare experiments have been reported which evaluate the biological activities of *H. rhodopensis* in vitro culture extracts.

Therefore, the current study aims to evaluate the biosafety and potential anti-ageing applications of *Haberlea rhodopensis* in vitro cell ethanol extract (HRT). Specifically, this study assesses the extract’s genotoxic, mitotoxic, and cytotoxic activities at varying concentrations. These assessments were conducted 24 and 72 h after treatment on a human embryonal cell line, Lep3. These findings are crucial for determining the viability of *H. rhodopensis* as a promising plant species in biomedical research.

## 2. Materials and Methods

### 2.1. Haberlea Rhodopensis Friv. In Vitro Culture Total Ethanol Extract (HRT)

Innova B.M. Ltd., Sofia, Bulgaria, generously provided *Haberlea rhodopensis* Friv. in vitro culture total ethanol extract (HRT) (https://innovabm.com/, accessed on 7 November 2023). The extract was obtained by maceration of *Haberlea rhodopensis* Friv. in vitro culture biomass with the water–ethanol mixture described elsewhere [20]. The provided extract was evaporated and then freeze-dried (Martin Christ, Alpha 1-2, Harz, Germany). The content of myconoside in the extract was analyzed by using HPLC Waters (1525 Binary pump and 2487 Dual λ Absorbance Detector) (Waters, Milford, MA, USA), with an injection volume of 20 µL, on column Supelco Discovery HS C18 (5 µm, 25 cm × 4.6 mm), operated at 28 °C with gradient of 2% acetic acid (A) and acetonitrile (B) as follows: 0–15 min 80% A; 15–17 min 50% A; 17–20 min 80% A. The concentration of myconoside in HRT was 181 ± 2.3 mg/g dry in vitro culture extract.

### 2.2. Cell Culture, Media and Treatment Protocols

The non-tumor human embryonic cell line Lep3 was used for the study experiments. The cells were grown in Dulbecco’s modified Eagle’s medium (DMEM) supplemented with 10% (*v*/*v*) fetal bovine serum (Sigma-Aldrich, Darmstadt, Germany) and 1% (*v*/*v*) HEPES (Gibco™/Thermo Fisher Scientific, Waltham, MA, USA). The cells were incubated at 37 °C in a fully humidified atmosphere at 5% CO_2_ in air. Media changes were performed twice a week. For cell experiments, the cells were detached with Trypsin-EDTA (Sigma-Aldrich, T4049) and plated at different densities depending on the experiment’s type in cell culture plates (Greiner bio-one, Cassina de Pecchi, Italy). After 24 and 72 h of incubation, Lep3 cells were treated with three concentrations of HRT extract: 25, 50, and 100 µg/mL (f.c.).

### 2.3. Phase Contrast Light Microscopy

Cell morphology alterations following a 24 and 72 h treatment with HRT extract with different concentrations were studied by phase-contrast microscopy. The micrographs were captured using a Carl Leitz microscope (Axivert 25, Jena, Germany) equipped with a digital camera at magnifications of 20×.

### 2.4. WST -1 Cell Viability Assay

The cytotoxicity assessment of HRT extract was performed using WST -1 assay (Sigma-Aldrich Co.) as previously described [21]. Initially, the cells were seeded into 96-well plates at a density of 2 × 10^4^ cells per well and allowed to incubate for 24 h at 37 °C and 5% CO_2_. On the next day, the culture medium was replaced with fresh medium, and the cells were exposed to increasing concentrations of the tested HRT extracts for an additional 24 and 72 h. At the end of incubation, the cell medium was removed and replaced with a new one. The WST -1 reagent was added directly to the cells in a ratio of 1:10, following the manufacturer’s instructions. After a 2 h incubation at 37 °C in darkness, the amount of the formazan produced by the cells was measured using a standard microplate reader (Thermo Scientific Multiskan Spectrum). The results were graphically presented with bars denoting the MEAN values ± STDV of three experiment repetitions. The cell proliferation data were normalized to the percentage of the untreated control. A Student’s *t*-test was applied for statistical data verification using Excel, Version 2016, explicitly utilizing the Data Analysis Toolpak for performing the calculations.

### 2.5. DCFH-DA Assay

For the determination of the ability of the HRT plant extracts to affect the ROS production inside Lep3 cells, a 2,7-dichlorodihydrofluorescein diacetate (DCFH-DA) assay was conducted. DCFH-DA, initially a non-fluorescent compound, penetrates the cell membrane and reacts with ROS, forming the highly fluorescent compound dichlorofluorescin (DCF). Briefly, Lep3 cells were incubated with various concentrations of plant extract (25, 50, and 100 µg/mL) to induce the antioxidant system in cells using bioactive compounds. After 24 and 72 h of incubation, cells were washed with phosphate buffer saline (PBS) and then incubated for 30 min in 20 μM DCFH-DA (Molecular Probes, Invitrogen, Basel, Switzerland) in the medium at 37 °C and 5% CO_2_. After the washing step with PBS, the cells were scraped. Fluorescence intensity was measured at an excitation wavelength of 485 nm and an emission wavelength of 528 nm using FP-8300 Fluorescence Spectrometer, Jasco Inc., Tokyo, Japan. Three repetitions of the experiment were done, and the data were statistically evaluated using a student’s *t*-test in Excel.

### 2.6. Single Cell Gel Electrophoresis (SCGE)

The SCGE procedure, also named comet assay, followed the previously established protocol [22]. Microgels were made by mixing the control cells and incubating them with the tested HRT concentrations for 24 and 72 h with 0.7% (*w/v*) low-gelling agarose (Sigma-Aldrich, Germany). Subsequently, the slides were subjected to lysis using a solution containing 146 mM NaCl, 30 mM EDTA (pH 7), 10 mMTris-HCl (pH 7), and 0.1% N-lauroylsarcosine (NLS, Sigma-Aldrich, Germany) for 20 min at 10 °C, followed by washing in 0.5xTBE buffer twice. Electrophoresis was then performed for 10 min at a voltage of 0.46 V/cm. The microgels were stained with SYBR green (molecular probes, Invitrogen) and examined under a fluorescent microscope. CometScore Version 1.5 software was used for analysis. The positive control for genotoxicity was Lep3 cells treated with 100 mM H_2_O_2_ for 15 min at 37 °C (Sigma-Aldrich). The experiment was conducted in triplicate, and the data were statistically analyzed using a Student’s *t*-test in Excel.

### 2.7. Fluorescence Activated Cell Sorting Analysis (FACS)

#### 2.7.1. Cell Cycle Analysis via Propidium Iodide (P.I.) Staining

Cell cycle analysis of Lep3 cells was conducted 24 h post-treatment with the described HRT extracts of the following concentrations: 25 µg/mL, 50 µg/mL, and 100 µg/mL. The cells were fixed using 96% cold ethanol and stored at −20 °C for 24 h. After fixation, the cells were collected by centrifugation, rinsed with PBS buffer, and treated with 100 µg/mL of RNAse A at 37 °C for 30 min. Subsequently, they were stained with 50 µg/mL of P.I. under dark conditions for 30 min. Cells were analyzed by flow cytometry, with red fluorescence detected at an excitation wavelength of 488 nm. FlowJo™ software Version 10 Ashland (Becton, Dickinson and Company; 2019, San Diego, CA, USA) was used to analyze the obtained data. Forward (FSC) and side scatter (SSC) acquisition were performed linearly. Three iterations of the experiment were performed, with data subjected to statistical analysis via a Student’s *t*-test using Excel.

#### 2.7.2. Analysis of Mitochondrial Membrane Potential (MMP) via Rhodamine 123 (Rh123) Staining and FACS

Flow cytometry analysis was conducted to assess the impact of the HRT extracts on mitochondrial activity in Lep3 cells, following the methodology described [21]. Mitochondria within individual cells were labelled with Rhodamine 123 (Rh123), a fluorescent dye commonly used to evaluate mitochondrial metabolism and activity [23,24,25,26]. In brief, adherent cells were gently scraped, washed, and resuspended in a pre-warmed complete DMEM medium at 37 °C. A negative control group was established by treating an aliquot of cells with 60 µM FCCP (mitochondrial inhibitor) at 37 °C for 20 min before Rh123 staining. Staining with 1 µg/mL of Rh123 was performed on all samples, which were then incubated for 30 min at 37 °C. Subsequently, the cells were centrifuged at 5000 rpm for 5 min at 4 °C, followed by washing with cold 1× PBS (pH 7.0) twice, being placed on ice, and immediately being subjected to flow cytometry analysis using a BD FACSCalibur™ instrument (Becton Dickinson, Hong Kong, China). Fifty thousand cells were acquired, and the data obtained were analyzed using FlowJo™ software, as described in the cell cycle progression experiments. The experimental procedures were carried out three times, and the resulting data were evaluated using a Student’s *t*-test in Excel for statistical validation.

### 2.8. Analysis of Mitochondrial Morphology via Biotraker 488 Staining and Fluorescent Microscopy

Cells were cultivated on circular glass coverslips in 2 mL DMEM, with each coverslip placed in an individual well from a 24-well plate. After a gentle wash with 2 mL DMEM, 1 mL of the culture media was removed. Cell fixation was carried out by adding 10 µL of 37% formaldehyde (0.37% HCHO) to the culture media and gently shaking to ensure even distribution. The cells were then incubated for 5 min at 37 °C. Following the incubation, dyes were added to the cells, including 2 µL of 1 mg/mL DAPI (resulting in a final concentration of 2 µg/mL in 1 mL DMEM) and 1 µL of 200 µM BioTracker 488 Green Mitochondria dye (Merck Bulgaria EAD, Sofia, Bulgaria) (resulting in a final concentration of 200 nM in 1 mL DMEM). Finally, the coverslip was carefully removed from the plate well using tweezers, and any excess liquid was absorbed by gently touching the edge of the coverslip to a paper towel or filter paper. The samples were examined using a fluorescent microscope by applying a tiny droplet of immersion medium.

## 3. Results

### 3.1. Biochemical Assessment of the HRT Extracts

**Figure 1:**
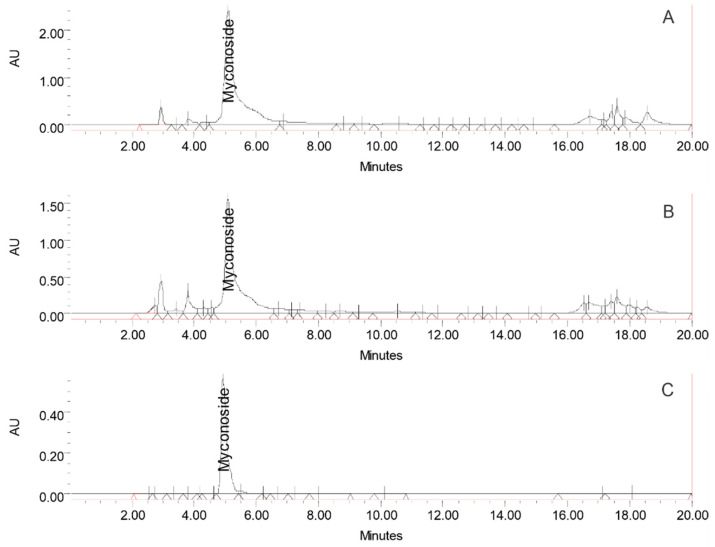
HPLC fingerprint of ethanol extracts from *H. rhodopensis* plant leaves (**A**), *H. rhodopensis* in vitro cultures (**B**), and myconoside standard (**C**).

The biochemical composition of the total ethanol extract from the in vitro culture of *H. rhodopensis* was analyzed using HPLC and compared with the extract from the plant leaves. The chromatograms illustrating both extracts are presented in Figure 1. The findings revealed a similar phytochemical profile between the total ethanol extract of the *H. rhodopensis* in vitro culture and that from the plant leaves. The dominant compound identified in both extracts was myconoside (181 ± 2.3 mg/g dry in vitro culture extract). Myconoside is a caffeoyl phenylethanoid glycoside identified as a significant molecule in the *Haberlea* plant and playing a crucial role in its survival [27].

The content of phenylethanoids in the total ethanol extract from the in vitro culture of *H. rhodopensis* was analyzed using HPLC and compared with the extract from the plant leaves. The HPLC fingerprints of pnenylethanoids found in both extracts are presented in Figure 1. The findings revealed similar profiles between the total ethanol extract of *H. rhodopensis* in vitro culture and that of the plant leaves, which differ only in the quantities of detected compounds. The dominant compound in both extracts was found to be myconoside. Its concentration in the investigated ethanol extract of *H. rhodopensis* in vitro culture was 181 ± 2.3 mg/g dry extract. It is well known that different extraction methods can lead to different accompanying compounds in the final extract. The HPLC profiles of phenolic compounds found in the total ethanol extract of *H. rhodopensis* in vitro culture and ethanol extract from *Haberlea* plant leaves are presented in Supplementary Figure S1. At the same time, the quantitation data are provided in Supplementary Table S1. A comparison between both extracts reveals significant differences in their chemical compositions. Notably, the extract from plant leaves contains rosmarinic acid, quercetin, and kaempferol, which are absent in the in vitro culture extract. This absence underscores the purity and richness of the HRT extract in myconoside, a critical active compound. The high concentration of myconoside in the in vitro HRT ethanol extract highlights its potential for specific therapeutic applications, including anti-ageing research.

### 3.2. Cytotoxicity Assessment of the HRT Extracts on the Lep3 Cell Line

**Figure 2:**
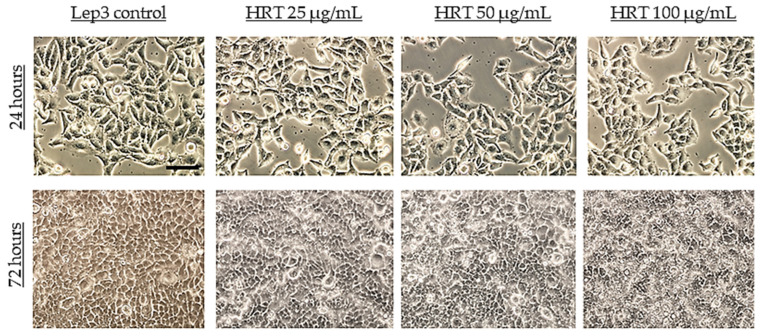
Representative phase-contrast micrographs illustrating morphology and density of Lep3 cells after 24 and 72 h of treatment with HRT extracts at concentrations of 25, 50, and 100 µg/mL (bar 100 µm).

To investigate the cytotoxic effect of HRT extracts on the normal human cell line Lep3, we conducted a series of experiments, including phase-contrast microscopy, standard WST-1, and DCFH-DA assay. These assays were performed with three various concentrations of the HRT extracts (25, 50, and 100 μg/mL) after 24 and 72 h of treatment. The objective was to analyze the morphological changes, quantify the cell viability and growth, and measure intracellular reactive oxygen species (ROS) levels, including hydroxyl radicals (•O.H.). The phase-contrast microscopy assessed the morphological changes in Lep3 cells treated with HRT extracts. Initially, Lep3 cells exhibited a typical cuboidal epithelial shape, firmly adhered to the tissue culture surface and appearing flattened, contacting, and spreading (Figure 2, upper panel). After 24 h of treatment with increasing concentrations of HRT extracts (25, 50, and 100 μg/mL), there were no noticeable changes in the cell morphology (Figure 2). However, a slight reduction in cell density was observed at higher concentrations. After 72 h of incubation, the cells treated with HRT extracts became more confluent than the control cells, suggesting a potential stimulatory effect on cell growth (Figure 2, lower panel). This increased confluency was observed at all tested concentrations, indicating that HRT extracts might enhance cell proliferation. The observations were made at a magnification of 20× to emphasize the overall monolayer confluency rather than individual cellular morphology.

**Figure 3:**
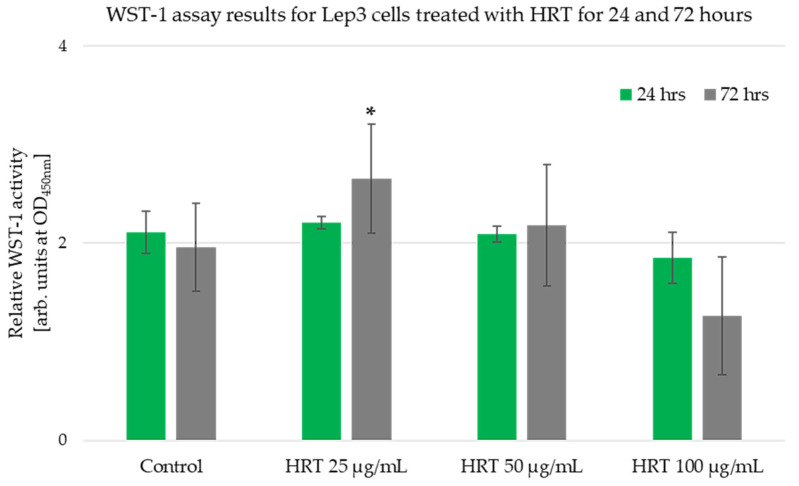
Cell viability of Lep3 cells after 24 and 72 h treatment with HRT extracts at 25, 50, and 100 µg/mL concentrations. Values are represented as MEAN ± STDV. The experiment was done in triplicate, and results were statistically evaluated; * denotes *p* < 0.005.

The observations of Lep3 cell morphology were consistent with the results obtained from the WST -1 assay (Figure 3). The WST-1 assay relies on the mitochondrial metabolic capacity of viable cells, and the measured values indicated that the HRT extract did not influence the metabolic activity or viability of human embryonic Lep3 cells within the examined concentration range of 25–100 μg/mL. Typically, a reduction in cell viability by more than 30% is considered a cytotoxic effect [28]. Our study showed no inhibition of cell viability in Lep3 cells due to treatment with HRT at the tested concentrations during the 24 h treatment period (Figure 3). The experiment was performed in triplicate, and there were no statistically significant differences in cell growth, as also visible on the micrographs in Figure 2 for this time interval. However, the treatment of the cells for 72 h revealed a statistically significant 40% increase in cell viability and growth at 25 μg/mL HRT. In contrast, the measured values for cells treated with 100 μg/mL were lower than the control. This decrease might be attributed to the very confluent cell layer and contact inhibition, likely causing the cells to die due to confluence.

Our findings show that the examined range of plant extract concentrations did not hamper cell growth and suggest the absence of metabolic inhibition and cytotoxicity. The results from the phase-contrast microscopy and WST-1 assay consistently suggest that HRT extracts do not exhibit cytotoxic effects on Lep3 cells at the concentrations tested. Instead, the extracts appear to enhance cell growth and proliferation. These findings highlight the potential of HRT extracts to support cell viability and growth in standard human cell lines.

**Figure 4:**
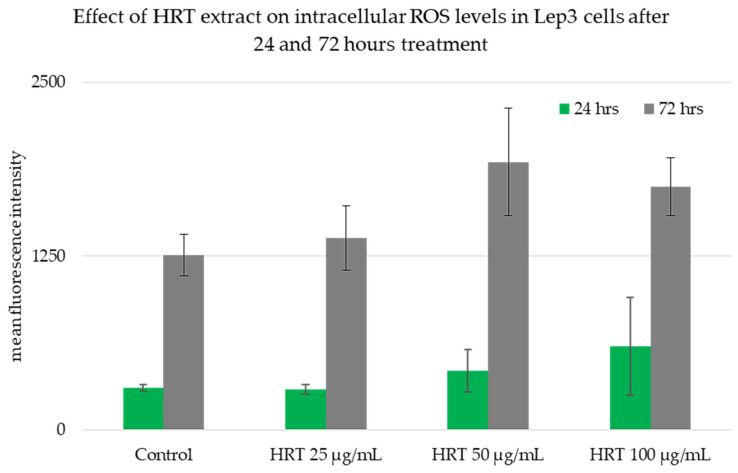
ROS production in Lep3 cells after treatment with HRT extracts at 25, 50, and 100 µg/mL concentrations for 24 and 72 h. Three repetitions of the experiments were done, and the detected differences were not significantly different.

The impact of HRT extract on the production of intracellular ROS levels in Lep3 cells was assessed through a cell-based assay using the DCFH-DA fluorescent probe (Figure 4). This nonpolar and non-ionic dye quickly diffuses into cells and is enzymatically converted by intracellular esterases into non-fluorescent 20,7-dichlorofluorescein (DCFH). In the presence of ROS, specifically intracellular oxidases and oxidants, DCFH is oxidized into the fluorescent 20,7-dichlorofluorescein (DCF), which remains inside the cells [29]. When the cellular antioxidant defense system does not compensate for cellular ROS production, oxidative stress can arise [30]. The quantification of ROS produced by Lep3 cells was assessed based on the fluorescence intensity. After 24 h, the ROS production was deficient in all samples, including both the control and HRT-treated cells. A slight increase in fluorescence intensity was detected in Lep3 cells treated with 50 µg/mL and 100 µg/mL HRT, though statistical analysis proved these differences were not significantly different (Figure 4). The same tendency was observed at 72 h post-treatment with HRT, where ROS production increased, as seen on the graph. Still, the differences were not significantly different again, which further supports the biosafety of HRT by demonstrating its lack of oxidative damage.

### 3.3. Genotoxicity Assessment of the HRT Extracts on the Lep3 Cell Line

**Figure 5:**
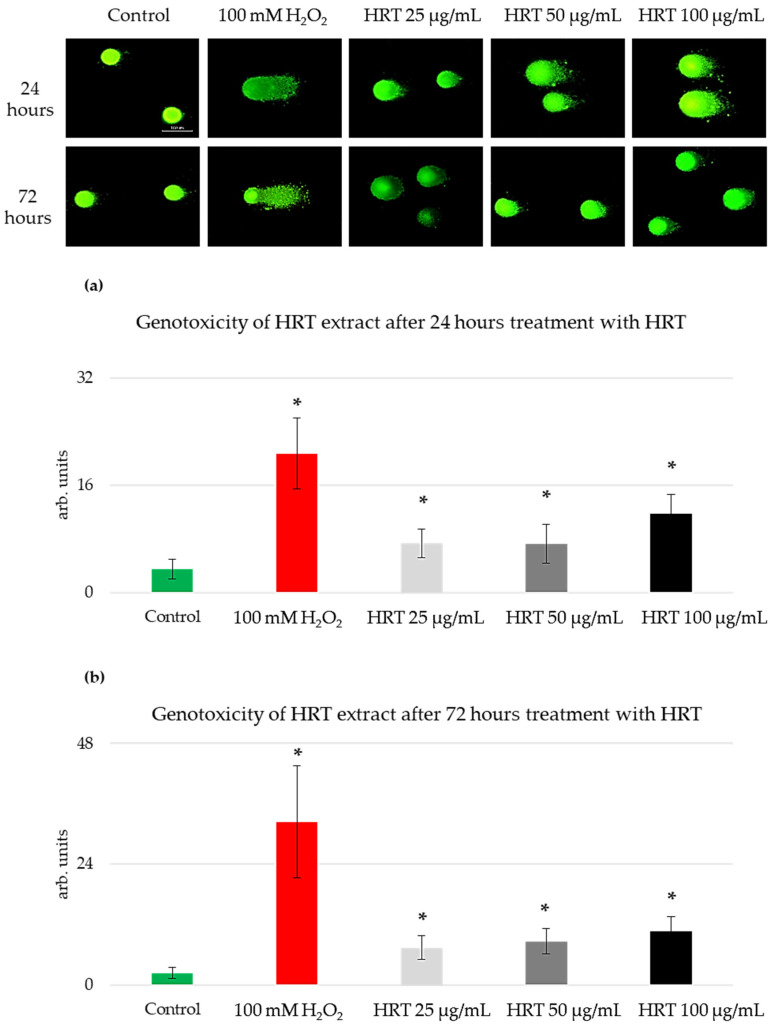
Investigation of the genotoxic potential of HRT extracts on Lep3 cells by the method of SCGE (comet assay). Representative comet assay images are presented on the upper panel. Values of the olive moment are represented as MEAN ± STDV. The experiment was conducted in triplicate, with statistical analysis applied to the results and * denoting *p* < 0.05. (**a**) 24 h treatment with HRT extracts; (**b**) 72 h treatment with HRT extract.

Having established that HRT extracts promote cell growth and proliferation in Lep3 cells without inducing morphological changes or oxidative stress, the next logical step was to assess the genotoxic potential of these extracts. While the previous assays confirmed the non-cytotoxic nature of HRT extracts and their stimulatory effects on cell growth, it is crucial to determine whether these extracts might cause DNA damage, which could have long-term implications for genomic stability. Therefore, we employed the comet assay, a sensitive technique for detecting DNA strand breaks at the single-cell level, to evaluate the genotoxic effects of HRT extracts on Lep3 cells. This method is a sensitive tool for genotoxic detection based on single-cell gel electrophoresis (SCGE) [31]. It is used to measure DNA strand breaks in individual cells. The underlying principle of the comet assay is that fragmented DNA moves from the nucleus and forms a tail when subjected to electrophoresis within an agarose gel. The procedure involves placing single-cell suspensions mixed with agarose onto microscope slides, lysing the cells using detergent and high-molarity NaCl to disrupt membranes, and subsequently subjecting the samples to electrophoresis. When DNA strand breaks are present, the DNA migrates towards the anode, resulting in a comet-like appearance when stained with a fluorescent dye and observed under fluorescence microscopy [31]. The assay’s versatility and sensitivity have made it a valuable tool for assessing DNA damage caused by various chemical or physical agents in cells derived from diverse organisms across various experimental conditions [32]. This method is frequently employed in human monitoring studies as a biomarker for evaluating exposure to agents that induce DNA damage [33]. The comet assay data were measured and analyzed using CometScore software [34]. The “Olive moment” parameter of the comet assay was utilized as an indicator of DNA damage and genotoxicity (Figure 5).

The Lep3 cells showed increased sensitivity to the used *H. rhodopensis* in vitro culture extract after 24 h of cultivation, proportional to the increasing concentrations of the extract, as depicted in Figure 5a. The olive moment values, which indicate genotoxicity, increased by two-fold for both 25 µg/mL and 50 µg/mL HRT extracts and three-fold for the 100 µg/mL HRT extract, compared to the control. The highest level of observed genotoxicity was noted in the cells treated with the highest extract concentration (100 µg/mL). In contrast, the increase in genotoxicity at the other two concentrations was very slight. For the positive genotoxicity control, cells were treated with 100 mM H_2_O_2_ for 15 min at 37 °C. This treatment resulted in significantly higher olive moment values than any of the HRT extract treatments, serving as a benchmark for substantial DNA damage. While all concentrations of HRT exhibited a slight genotoxic effect, none exceeded the genotoxicity observed in the positive control. The genotoxicity levels for the cells treated with HRT for 72 h were similar (Figure 5b). The genotoxicity by the extracts was considered insignificant compared to the positive control, suggesting that, although there was a measurable increase in DNA damage with higher concentrations of HRT extracts, it did not reach a level of concern.

### 3.4. Cytostatic Effect of the HRT Extract on Lep3 Cells

While the comet assay results indicated a slight genotoxic effect of HRT extracts at higher concentrations, it is essential to understand further how these extracts influence cellular processes such as the cell cycle. DNA damage can lead to alterations in cell-cycle progression, potentially causing cell-cycle arrest or changes in the distribution of cells across different phases. To understand the impact of HRT extracts on cell-cycle dynamics, we employed fluorescence-activated cell sorting (FACS) analysis. This technique allowed us to assess the distribution of Lep3 cells across various cell-cycle phases following treatment with HRT extracts, providing deeper insights into their potential effects on cell proliferation and genomic stability.

**Figure 6:**
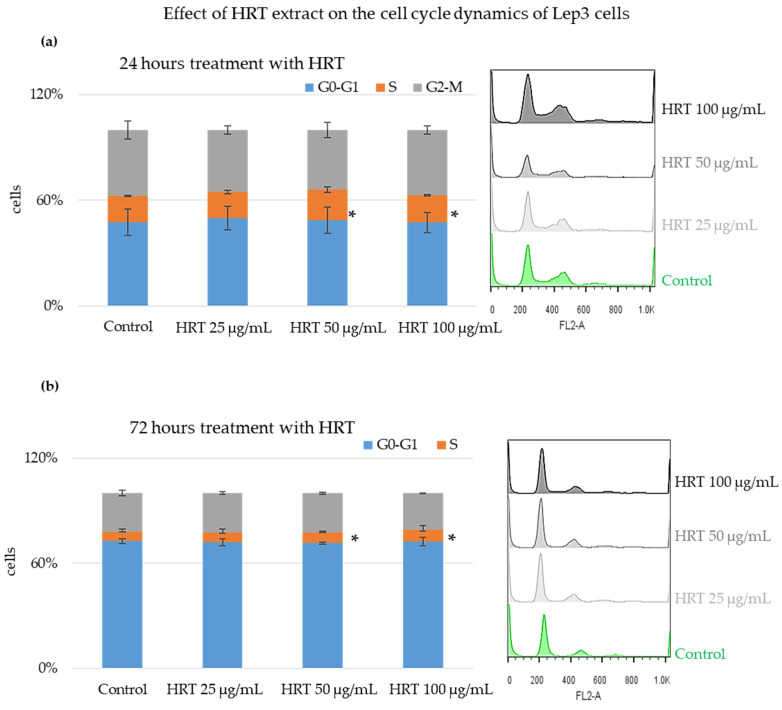
Cell cycle analysis of Lep3 cells treated with HRT extracts. Bar charts are presented with the percentage of cells in each cell-cycle phase. Experiments were carried out in triplicate, and the results were statistically evaluated. Values are represented as MEAN ± STDV. Representative histograms are provided on the right panels for each time point. (**a**) 24 h of cultivation; (**b**) 72 h of cultivation. Triplicates were performed; * denotes *p* < 0.05.

After staining with Propidium Iodide (PI), the Lep3 cells underwent FACS analysis to evaluate their progression through different cell-cycle phases. The results of the FACS analysis, presented as the percentage of cells in each cell cycle phase, are displayed in Figure 6. Figure 6a shows bar charts with the percentage of cells in each cell-cycle phase for all HRT extract concentrations after 24 h of cultivation, and Figure 6b shows the results after 72 h of cultivation. Both graphs have embedded representative histograms. The values are represented as MEAN ± STDV, and statistically significant differences compared to the control are indicated (* *p* < 0.05). Cells treated for 24 h with the HRT extracts showed a slight increase in the percentage of cells in the S phase, with approximately a 1.5-fold increase for both the 50 µg/mL and 100 µg/mL HRT concentrations. Triplicates were performed, and statistical analysis showed that these differences in the percentage of cells in the S phase upon treatment with the two HRT concentrations (50 and 100 µg/mL) were statistically significant. For the cells treated for 72 h, there was a predominant accumulation in G0-G1, indicative of cells at full confluency, as already seen in Figure 1 and Figure 2. However, at 50 and 100 µg/mL of the extract, there was a slight increase in the percentage of cells in the S phase, which proved statistically significant.

**Figure 7:**
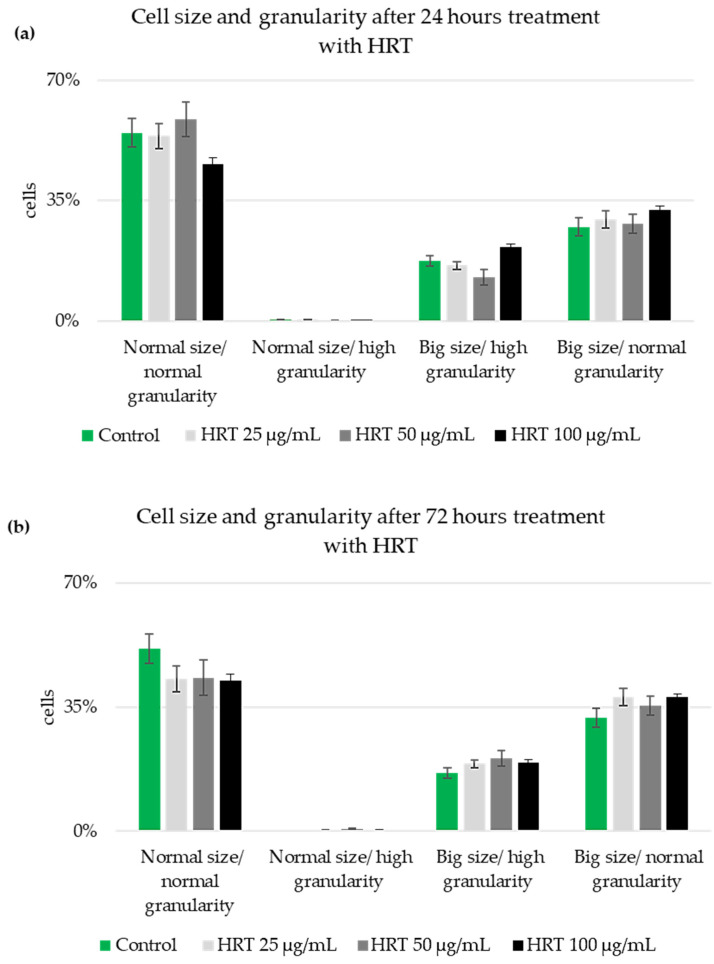
Cell scatter analysis of Lep3 cells, control and treated for 24 h with 25 µg/mL, 50 µg/mL, and 100 µg/mL of HRT extract, represented as cell percentage. Values are represented as MEAN ± STDV. (**a**) 24th hour of cultivation, (**b**) 72nd hour of cultivation. Experiments were carried out in triplicate, and the results were statistically evaluated.

The findings from the cell-cycle evaluation were further supported by analyzing the distribution of cells based on forward and side scatter parameters (FSC/SSC), which measure cellular size versus cellular granularity, as presented in Figure 7. After 24 h of treatment, no significant differences in cellular size or granularity were observed between the HRT-treated and untreated control cells across all tested concentrations (Figure 7a; 25 µg/mL, 50 µg/mL, and 100 µg/mL). These results indicate that the HRT extract did not noticeably affect the cell morphology within this time frame.

However, after 72 h of treatment, the percentage of cells with standard size and granularity was slightly reduced compared to the control (Figure 7b). This reduction might be attributed to increased cell growth, as indicated in Figure 2. These observations suggest that prolonged exposure to the HRT extract could lead to subtle changes in cell morphology due to enhanced cellular proliferation.

### 3.5. Mitochondrial Membrane Potential and Morphology Analyses

#### 3.5.1. Mitochondrial Membrane Potential Detection by Staining of Cells with Rh123 and FACS Analyses

**Figure 8:**
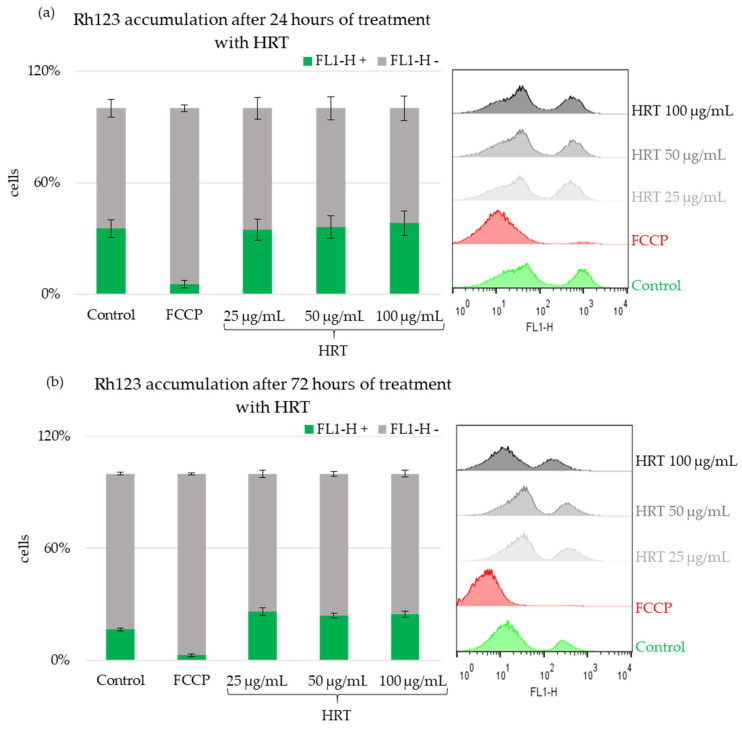
The impact HRT extract of 25 µg/mL, 50 µg/mL, and 100 µg/mL of HRT extract on mitochondria of Lep3 cells was evaluated using Rhodamine 123 (Rh123) staining and observed through FACS. Mitochondrial activity was assessed based on the uptake of Rh123 fluorescent dye, which is sensitive to changes in the mitochondrial membrane potential in viable cells. The fluorescence data were obtained using the FL1-H detector of the flow cytometer and represented as a percentage of cells with active MMP, with accompanying histograms. The experiments were done in triplicate, and the results underwent statistical analysis. (**a**) 24th hour of cultivation, (**b**) 72nd hour of cultivation.

To investigate the impact of various concentrations of the HRT extract on mitochondria, we employed Rhodamine 123 (Rh123) staining, a fluorescent dye known for its specific affinity to mitochondria based on their transmembrane potential in living cells. Subsequently, flow cytometry analysis was conducted to examine the Rh123 fluorescence in Lep3 cells treated with the HRT extract. The results of these experiments are presented in Figure 8, with data quantitation for the Lep3 cells after 24 h of cultivation shown in Figure 8a and the corresponding histograms displayed as well. Cell aliquots were pre-treated with FCCP to disrupt the mitochondrial membrane potential (MMP) for the negative control group, which exhibited impaired mitochondrial function. As expected, FCCP effectively hindered Rh123 uptake, indicating disrupted MMP and impaired mitochondrial respiratory function.

In the cells treated with HRT for 24 h, no significant differences in MMP were observed, as shown in Figure 8a. However, after 72 h of treatment with HRT, an induction of MMP was noted, with approximately a 1.6-fold increase in Rh123 fluorescence, irrespective of the concentration of the extract, as depicted in Figure 8b. The uptake of Rhodamine 123 measured via flow cytometry (FACS) indicates the mitochondrial membrane potential; higher fluorescence intensity corresponds to higher MMP, reflecting enhanced mitochondrial activity or increased mitochondrial mass. These findings suggest that, while short-term exposure (24 h) to the HRT extract does not affect mitochondrial function, prolonged exposure (72 h) may boost mitochondrial activity in Lep3 cells, as evidenced by the increased Rh123 fluorescence.

#### 3.5.2. Mitochondrial Structure Visualization by Biotracker 488 Staining

**Figure 9:**
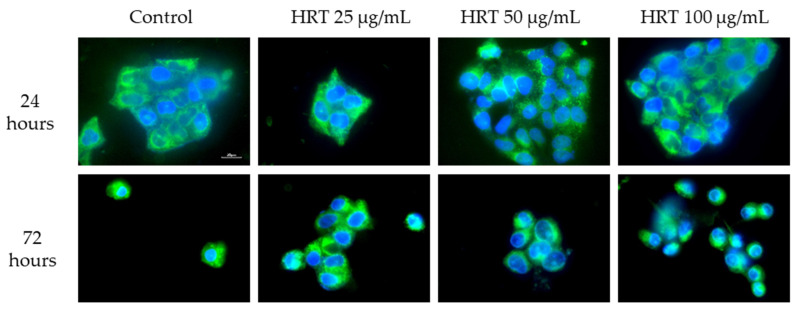
Fluorescence microscopy of mitochondrial structure in Lep3 cells, control and incubated with HRT extract concentrations with 25 µg/mL, 50 µg/mL, and 100 µg/mL. Representative epifluorescence images after staining with Biotracker 488 and DAPI are displayed. Scale bar, 20 µm.

To further assess the mitochondrial integrity, Lep3 cells were subjected to Biotracker 488 staining and observed under an epi-fluorescent microscope after treatment with varying concentrations of the HRT extract. Concurrently, DAPI staining was employed for nucleus visualization as part of the double-staining procedure. Figure 9 displays representative images, where a robust green fluorescence signal denotes preserved mitochondrial membrane integrity, notably enhanced in cells treated with the extract. This observation aligns with the Rh123 staining results, suggesting a favorable impact of the HRT extract on the mitochondrial membrane potential (MMP) of Lep3 cells across the tested concentrations after 72 h of treatment with HRT. Regardless of the treatment intervals, we observed a lack of mitotoxicity by the extracts. Some specific dotted-type morphologies of mitochondria were detected in cells treated with 50 µg/mL HRT, but this needs further investigation.

These findings contribute to our understanding of the biological effects of the HRT extract on cellular health, particularly in the context of mitochondrial function. Given the central role of mitochondria in various cellular processes, including ageing and disease development, the ability of the HRT extract to preserve mitochondrial integrity and function underscores its potential as a valuable candidate for therapeutic and anti-ageing interventions. Therefore, further research and clinical trials are warranted to explore the full potential of HRT extract in mitigating age-related cellular decline and its applications in medical treatments.

## 4. Discussion

Recently, *H. rhodopensis* has captured the scientific community’s interest as a plant with potential anti-ageing agent capabilities of its extracts due to its potent antioxidant properties and ability to enhance the synthesis of extracellular matrix proteins in the skin [35,36]. Scientific evidence also confirms the anti-inflammatory effects of extracts [37] and their protective effects against cellular damage induced by gamma irradiation [38]. In our current study, we analyzed the biosafety of the total ethanol extract of *H. rhodopensis* derived from fast-growing in vitro cultures in normal Lep3 cells, going deeper into its genotoxicity and cytotoxicity mechanisms of action. The studied total ethanol extract from the in vitro culture biomass of *H. rhodopensis* has been evaporated and freeze-dried to remove any residues of the solvent. Research indicates that organic solvents can have varying detrimental effects on cellular function [39]. For instance, in the cosmetics industry, pure ethanol has been observed to suppress collagen biosynthesis in fibroblast cultures [37]. Generally, ethanol toxicity can lead to various complications, including diminished respiratory function, which may progress to respiratory failure, as well as conditions such as hypothermia, cardiac dysrhythmias leading to cardiac arrest, hypoglycemia, ketoacidosis, and hypotension [35].

### 4.1. H. rhodopensis Extracts Demonstrate High Viability and Low Cytotoxicity in Lep3 Cells

HPLC fingerprints demonstrated that the phytochemical profile of the total ethanol extract of *H. rhodopensis* in vitro culture showed high similarities to that of the ethanol extract from *H. rhodopensis* plant leaves. The dominant compound in both extracts was identified as myconoside. Its concentration in the investigated ethanol extract of *H. rhodopensis* in vitro culture was 181 ± 2.3 mg/g dry weight. This indicates that the in vitro-cultured *H. rhodopensis* is a valuable source of biologically active metabolites, with no compromise in the expression of these metabolites during in vitro culturing. As evaluating plant extracts for potential cytotoxicity is crucial for assessing their suitability for further applications [37], we initially assessed the effects of the total ethanol extract of *H. rhodopensis* in vitro culture on the viability and morphology of Lep3 embryonic cells. Our findings revealed the non-toxic nature of the *H. rhodopensis* total extract towards Lep3 cells. Further, no oxidative damage was detected as, typically, ROS-induced oxidative stress can inflict damage on cellular biomolecules, including DNA, proteins, and lipids, potentially compromising essential cellular functions such as metabolic activity, and causing the accumulation of harmful mutations, genome instability, cancer, accelerated cellular senescence, and even cell death. However, our findings demonstrated that even treatment with the highest concentration of *H. rhodopensis* extract for 24 and 72 h did not induce any statistically significant ROS production. Thus, based on the results obtained from the cytotoxicity assessment, it can be inferred that the HRT total extract could positively influence normal Lep3 cells when applied at appropriate concentrations. This contrasts with some previously reported results demonstrating a toxicity effect of HRP plant extract in 3T3 mouse embryonic cells. The observed effect is probably because we used *H. rhodopensis* in vitro cultures extract (HRT), which contains significantly fewer amounts of concomitant metabolites such as rosmarinic acid and kaempferol than the ethanol extract described in the previous study [40] (Figure S1 and Table S1). The dose-dependent cytotoxic effects of rosmarinic acid and kaempferol on some cell lines have been reported previously [41,42].

### 4.2. Non-Genotoxic Effects of Low-Dose Haberlea rhodopensis In Vitro Culture Extracts

Detecting genotoxicity in medicinal plant extracts is a crucial step, as it is directly relevant to human safety, potentially impacting carcinogenesis and hereditary defects. Single-cell gel electrophoresis, commonly known as comet assay, is a widely utilized method for detecting DNA damage at the single-cell level. In our experiments, the neutral form of the assay primarily identified double-stranded DNA breaks, indicative of DNA damage resulting from apoptosis [37]. We tested the *H. rhodopensis* in vitro culture extract at 25 µg/mL, 50 µg/mL, and 100 µg/mL for potential genotoxic effects on the normal fibroblast cell line Lep3 over 24 and 72 h. The positive control for genotoxicity was cells treated with H_2_O_2_, and, compared to them, our data with the HRT treatment for 24 and 72 h confirmed no statistically significant genotoxic effects in the cells treated with the extracts. This finding is promising, as it suggests that the HRT extract can be safely utilized at these concentrations for various biomedical and cosmetic applications without eliciting adverse genotoxic effects.

### 4.3. Enhanced Mitochondrial Activity and Cell Cycle Distribution in Lep3 Cells Treated with H. rhodopensis In Vitro Extracts for More Extended Periods

The cells treated with the extract were additionally examined for cytostatic stress. After treating Lep3 cells with the three varying concentrations, a FACS analysis was conducted to analyze the cells’ distribution across different cell-cycle phases. The tested concentrations of *H. rhodopensis*, 25 µg/mL, 50 µg/mL, and 100 µg/mL, for 24 and 72 h of treatment showed no cytostatic effect. There was no evidence of cells being blocked in any cell-cycle phase. This outcome further confirms the extract’s biocompatibility with normal human fibroblast cells.

Mitochondria, cellular organelles abundant in most cells, play a vital role in executing essential biochemical processes such as respiration and energy production [38]. In cells with high metabolic activity, mitochondria establish a membrane potential by maintaining a proton gradient across their inner and outer membranes. A decline in mitochondrial membrane potential is a hallmark of apoptosis and signifies compromised cellular health [37]. The mitochondrial activity and morphology were assessed using two distinct methods: Rhodamine 123 (Rh123) staining to detect mitochondrial membrane potential activity and staining with BioTracker 488/DAPI to observe the mitochondrial morphology. Two time points were evaluated: 24 and 72 h. The *H. rhodopensis* in vitro extracts favorably affected the mitochondrial activity in Lep3 cells after the extended treatment period. The findings indicate a slight increase in mitochondrial potential, further supported by fluorescent microscopy observations that revealed an enhanced mitochondrial morphology resembling mitochondrial fission, indicative of cellular rejuvenation [37].

## 5. Conclusions

In conclusion, the significance of our study is underscored by the unique status and conservation needs of *H. rhodopensis*. It is protected as a rare species with limited populations in specific geographical locations, and collecting individual plants or parts from its natural habitats is strictly forbidden. Traditional propagation methods, such as micropropagation and alternative propagation in hydroponic systems and greenhouses, have proven slow and economically inefficient, with a low commercialization potential. However, recent biotechnological advancements have enabled the renewable production of *H. rhodopensis* biomass through the large-scale cultivation of differentiated and undifferentiated in vitro cultures. These cultures demonstrate rapid growth and accumulate secondary metabolites at higher concentrations than the mother plants. Growing *H. rhodopensis* in vitro cultures under controlled conditions is currently the most viable method for the sustainable large-scale production of rare metabolites such as myconoside. Despite this progress, few experiments have evaluated the biological activities of *H. rhodopensis* in vitro culture extracts. Our study addresses this gap by assessing mitochondrial activity and morphology, providing crucial insights into the potential therapeutic applications of these extracts. The provided comprehensive analysis demonstrated that the *H. rhodopensis* in vitro culture extract showed no cytotoxicity or genotoxicity at lower concentrations and enhanced the mitochondrial activity and morphology in Lep3 cells after extended treatment periods. These findings underscore the extract’s promising role in the medical and cosmetic sectors. This study highlights the safety considerations and therapeutic potential of *H. rhodopensis* in vitro culture extract, paving the way for further exploration into its mechanisms of action and broader applications in health and wellness.

Key takeaways include the extract’s compatibility with human cells, its positive effects on cellular rejuvenation, and its potential as a valuable resource for future biomedical research and development efforts.

## Data Availability

Data are available upon request.

## Supplementary Materials

The following supporting information can be downloaded at: https://www.mdpi.com/article/10.3390/cells13131118/s1, Figure S1. HPLC chromatograms (360 nm) of ethanol extracts from *H. rhodopensis* plant leaves HRP (A) and *H. rhodopensis* in vitro cultures HRT (B). The used HPLC method is described elsewhere; Table S1. HPLC determination of rosmarinic acid, quercetin and kaempherol in ethanol extracts from *H. rhodopensis* plant leaves HRP and *H. rhodopensis* in vitro cultures HRT.
