# Supplementary material for: Exploring the Biosafety Potential of Haberlea rhodopensis Friv. In Vitro Culture Total Ethanol Extract: A Comprehensive Assessment of Genotoxicity, Mitotoxicity, and Cytotoxicity for Therapeutic Applications
Source: Cells. 2024 Jun 28;13(13):1118. doi: 10.3390/cells13131118 (PMC11240332; doi:10.3390/cells13131118)
Supplement: Supplementary file 1 [file cells-13-01118-s001.zip › cells-3002490-supplementary.pdf]

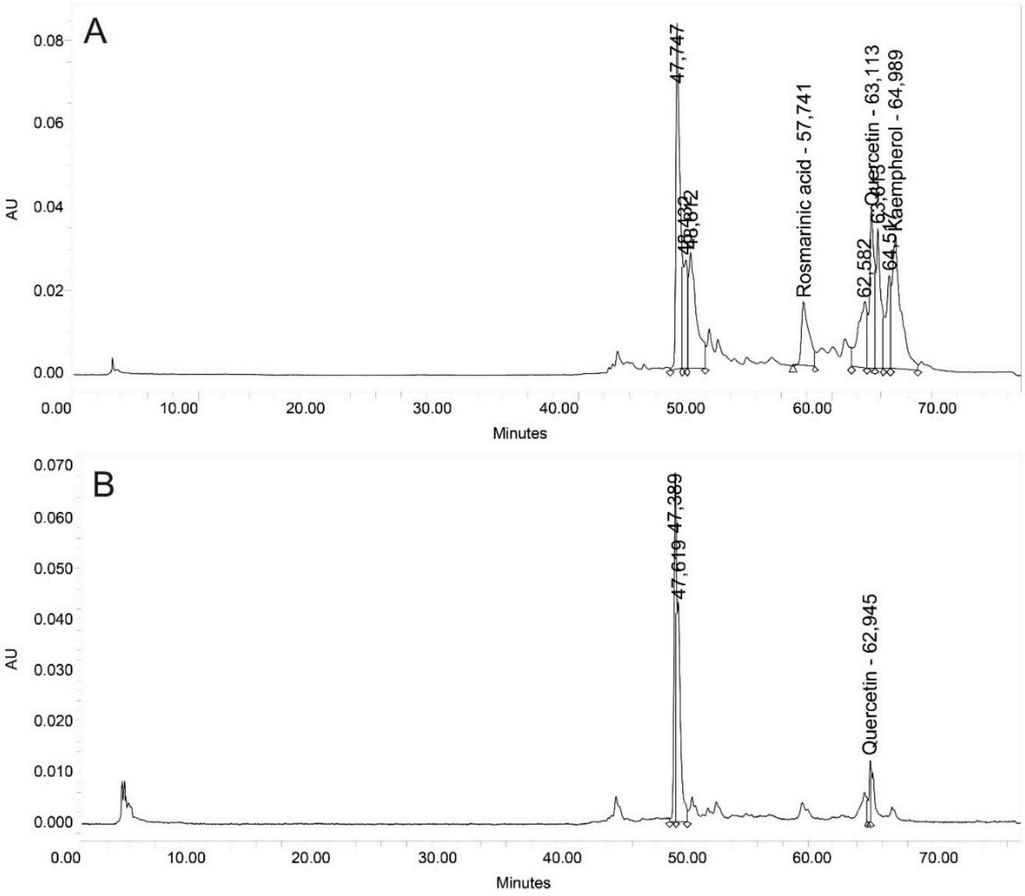

Figure S1. HPLC chromatograms (360 nm) of ethanol extracts from *H. rhodopensis* plant leaves HRP (A) and *H. rhodopensis* in vitro cultures HRT (B). The used HPLC method is described elsewhere (Krasteva et al. 2022; https://doi.org/10.3390/molecules27248906).

Table S1. HPLC determination of rosmarinic acid, quercetin and kaempherol in ethanol extracts from *H. rhodopensis* plant leaves HRP and *H. rhodopensis* in vitro cultures HRT.

Compounds	*H. rhodopensis* plant leaves HRP, mg/g Dry Extract	*H. rhodopensis in vitro* cultures HRT mg/g Dry Extract
Rosmarinic acid	3.15 ± 0.12	NF *
Quercetin	0.78 ± 0.02	ULOQ **
Kaempferol	1.23 ± 0.11	NF

*-Not Found, **-Under The Limit of Quantification.

References

Krasteva, G.; Berkov, S.; Pavlov, A.; Georgiev, V. Metabolite Profiling of *Gardenia jasminoides* Ellis In Vitro Cultures with Different Levels of Differentiation. *Molecules* **2022**, *27*, 8906. https://doi.org/10.3390/molecules27248906
